# A case of cryopyrin-associated periodic syndrome presenting with abdominal symptoms due to a novel mutation *NLRP3* p.Ile257Met

**DOI:** 10.1007/s12328-026-02293-5

**Published:** 2026-03-17

**Authors:** Yo Komatsu, Tomofumi Oizumi, Shun-ichi Yanai, Yumiko Kobayashi, Shin-ya Nishio, Shin-ichi Usami, Takayuki Matsumoto

**Affiliations:** 1 Division of Gastroenterology, Department of Internal Medicine, School of Medicine, Iwate Medical University, Idaidori 1-1-1, Yahaba, Shiwa, 028-3694 Japan; 2 Department of Clinical Genetics, School of Medicine, Iwate Medical University, Shiwa, Japan; 3 Department of Hearing Implant Sciences, Shinshu University School of Medicine, Matsumoto, Nagano Japan

**Keywords:** Cryopyrin-associated periodic syndrome, NLRP3, Canakinumab

## Abstract

A 24-year-old female patient with a 4-year history of recurrent abdominal pain, diarrhea, periodic fever and elevated C-reactive protein was referred to our institution. Colonoscopy, esophago-gastro-duodenoscopy, capsule endoscopy and contrast-enhanced CT showed no significant findings. Because she and her mother had sensorineural hearing loss, a diagnosis of cryopyrin-associated periodic syndrome was suspected. Genetic testing of the patient and her mother revealed a candidate germline variant of *NLRP3*. The patient was commenced on subcutaneous 150 mg canakinumab, a fully human monoclonal antibody targeting IL-1β, at a dose of 150 mg every 8 weeks, which resulted in the persistence of sensorineural hearing loss but led to the complete disappearance of her abdominal symptoms and periodic fever.

## Introduction

Cryopyrin-associated periodic syndrome (CAPS) is an autoinflammatory disease with autosomal dominant inheritance [1]. It is a rare disease with an estimated prevalence of 1 to 2 per 1 million [2]. CAPS has three clinical phenotypes, namely, familial cold autoinflammatory syndrome (FCAS), Muckle-Wells syndrome (MWS) and chronic neonatal onset multisystem inflammatory disorder (CINCA/NOMID). While those three diseases were considered to be clinically distinctive, the discovery of common causal variants in *NLRP3* (Nucleotide-binding oligomerization domain-like receptor family, pyrin domain-containing 3) gene has led to the integration of these three diseases into an entity known as CAPS [1]. Indeed, the three phenotypes are often overlapping [2–4].

Although the prevalence of gastrointestinal (GI) symptoms in CAPS is less common compared with that in familial Mediterranean fever (FMF), which is another type of hereditary autoinflammatory disease, it is noted that low-penetrance *NLRP3* variants can sometimes induce fever and severe GI symptoms mimicking inflammatory bowel disease (IBD) [5]. We herein report on a patient with CAPS who presented with recurrent fever, hearing loss, abdominal pain and diarrhea. Our experience suggested that CAPS is a differential diagnosis of patients suspected of having monogenic IBD.

## Case report

A 20-year-old female was admitted to the neighboring hospital due to recurrent abdominal pain, a mixture of intermittent diarrhea and constipation, and periodic fever for several days per month. On some occasions, exposure to cold circumstances such as an air conditioner triggered abdominal pain. She also had a prior and ongoing history of sensorineural hearing loss since the age of 10. She had not experienced chronic arthritis or ocular manifestations such as conjunctivitis or uveitis. Colonoscopy and plain CT revealed no significant findings. Four years after the symptom onset, at the age of 24, she was referred to our institution for further assessment of the small bowel with a suspicion of Crohn’s disease.

**Fig. 1:**
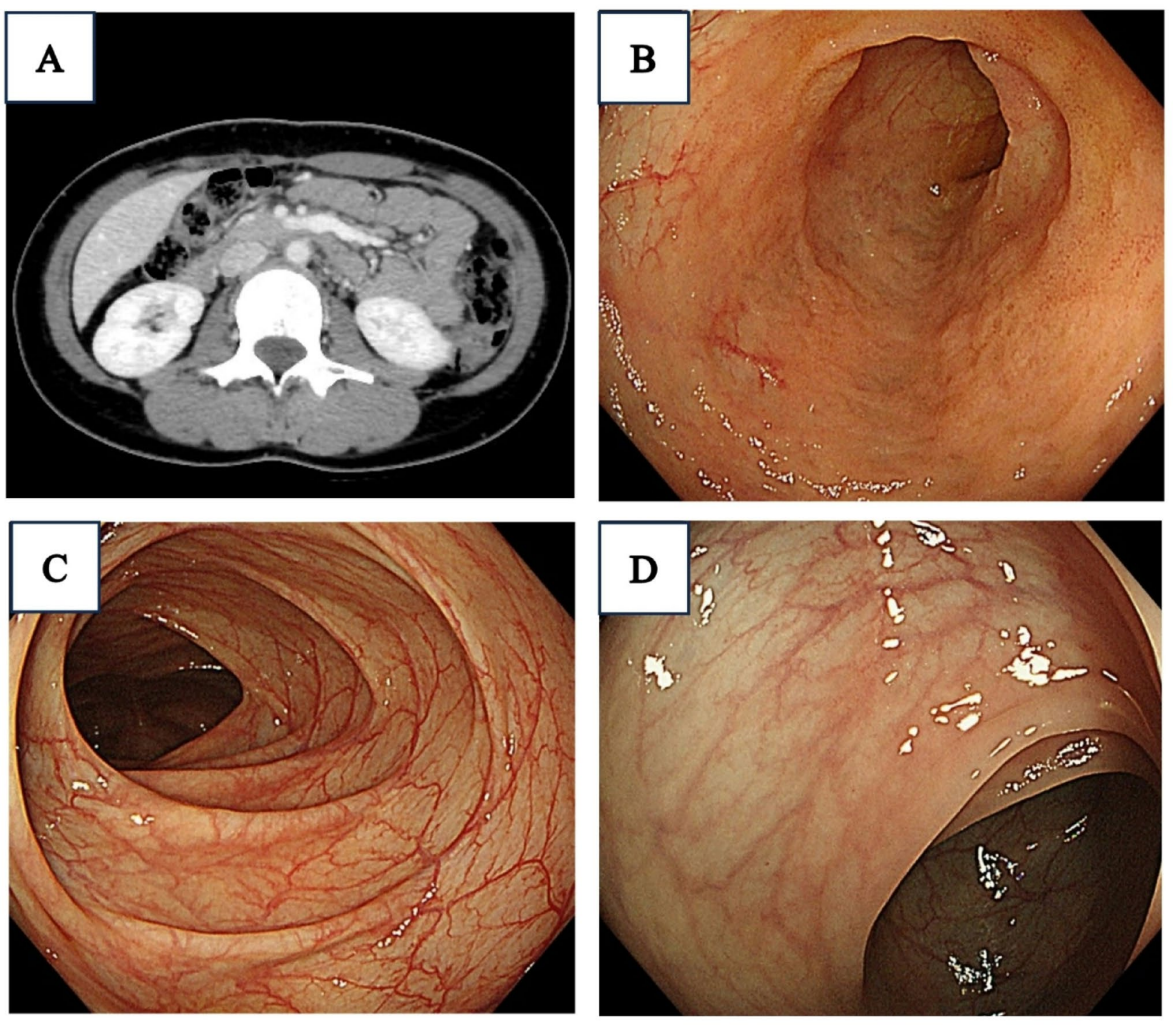
Clinical images of this case: **A** abdominal CT scan, **B** colonoscopy for terminal ileum, **C** colonoscopy for ascending colon, and **D** colonoscopy for rectum

At the time of admission, she was asymptomatic and afebrile. Physical examination was unremarkable without any perianal lesions. Laboratory findings showed mild anemia, leukocytosis with predominant neutrophils, and an increase in C-reactive protein (CRP) value (2.27 mg/dL) (Table 1). Colonoscopy and esophago-gastro-duodenoscopy (EGD) together with histological evaluation of biopsy specimens, as well as capsule endoscopy and contrast-enhanced CT, revealed no significant findings suggestive of Crohn’s disease or other IBD (Fig. 1). Based on these findings, we made a tentative diagnosis of familial Mediterranean fever (FMF). According to Tel-Hashomer criteria [6], her FMF was classified as typical attacks, as indicated by periodic high fever lasting several days per month, along with abdominal pain. However, genetic testing of *MEFV* gene did not identify any pathogenic variants. Whereas we administered oral colchicine therapy (0.5 mg/day), she continued to manifest periodic fever and abdominal pain.

**Table 1:** The initial laboratory findings

Hematology		Blood chemistry			
WBC	12,730 /µL	Na	138 mEq/L	IgG	1464 mg/dL
Neutro	80.5%	K	4.2 mEq/L	IgA	711 mg/dL
Lymph	12.9%	Cl	105 mEq/L	IgM	182 mg/dL
Eosino	1.8%	BUN	13.0 mg/dL	CEA	0.6 ng/mL
Baso	0.20%	Cre	0.38 mg/dL	CA19-9	16.5 U/mL
Mono	3.70%	AST	11 U/L	CH50	> 60 U/mL
RBC	466 × 10^4^ /µL	ALT	10 U/L	C3	149 mg/dL
Hb	10.8 g/dL	ALP	88 U/L	C4	26 mg/dL
Hct	34.4%	γ-GTP	15 U/L	ANA	(-)
Plt	31.7 × 10^4^ /µL	T-Bil	0.4 mg/dL	RF	(-)
		LDH	91 U/L	RNP Ab	(-)
		TP	8.1 g/dL	PR3-ANCA	(-)
		Alb	4.1 g/dL	MPO-ANCA	(-)
		CK	21 U/L		
		CRP	2.27 mg/dL		

WBC, white blood cell; Neutro, neutrophil; Lymph, lymphocyte; Eosino, eosinophil; Baso, basophil; Mono, monocyte; RBC, red blood cell; Hb, hemoglobin; Ht, hematocrit; Plt, platelet; BUN, blood urea nitrogen; Cre, creatinine; AST, aspartate aminotransferase; ALT, alanine aminotransferase; ALP, alkaline phosphatase; γ-GTP, γ-glutamyl transpeptidase; T-Bil, total bilirubin; LDH, lactate dehydrogenase; TP, total protein; Alb, albumin; CK, creatine kinase; CRP, C-reactive protein; IgG, immunoglobulin G; IgA, immunoglobulin A; IgM, immunoglobulin M; CEA, carcinoembryonic antigen; CA19-9, carbohydrate antigen 19 − 9; CH50, 50% hemolytic complement activity; C3, complement 3; C4, complement 4; ANA, antinuclear antibody; RF, rheumatoid factor; RNP Ab, anti-ribonucleoprotein antibody; PR3-ANCA, proteinase 3 antineutrophil cytoplasmic antibody; MPO-ANCA, myeloperoxidase antineutrophil cytoplasmic antibody

**Fig. 2:**
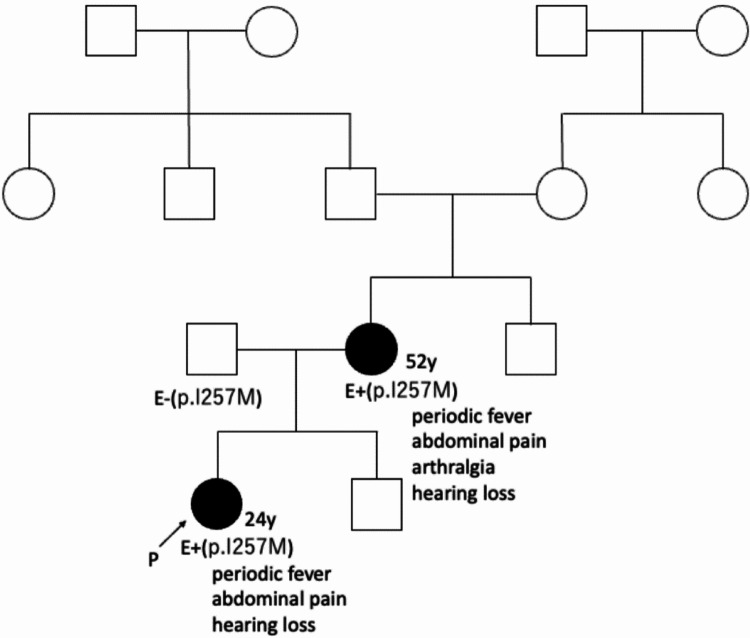
Pedigree tree of the family

Thereafter, it became evident that her mother had also been suffering from sensorineural hearing loss since her childhood. Also, she had a history of periodic fever, abdominal pain, and arthralgia. Her father, grandmother, grandfather, uncle and aunt had never exhibited similar symptoms. The family pedigree of the patient is indicated in Fig. 2. Based on these findings, a diagnosis of cryopyrin-associated periodic syndrome (CAPS), rather than FMF, was suggested. Subsequent genetic testing for *NLRP3* gene revealed that both the patient and her mother carried a heterozygous variant of *NLRP3*, while her father, who did not exhibit any symptoms, did not carry this variant. The identified variant was *NLRP3*:NM_004895.4:c.771 C > G and estimated to cause p.Ile257Met (p.I257M) missense mutation with amino acid substitution. These findings confirmed a diagnosis of MWS, the phenotype of which was compatible with CAPS.

We started to treat the patient with subcutaneous administration of canakinumab, a fully human monoclonal antibody targeting IL-1β, at a dose of 150 mg every 8 weeks. The treatment did not improve her sensorineural hearing loss but it led to amelioration of the abdominal symptoms, periodic fever and CRP levels (Table 2). While serum amyloid A (SAA) level was not measured prior to the treatment, it was continuously within the normal limit thereafter. She has been remaining asymptomatic and afebrile. Her mother is also now considered to administer canakinumab.

**Table 2:** The laboratory findings 30 days after the first administration of Canakinumab

Hematology		Blood chemistry			
WBC	5620 /µL	Na	138 mEq/L	T-Bil	0.6 mg/dL
Neutro	51.2%	K	4.3 mEq/L	LDH	132 U/L
Lymph	39.2%	Cl	103 mEq/L	TP	7.8 g/dL
Eosino	1.8%	BUN	11.5 mg/dL	Alb	4.5 g/dL
Baso	0.30%	Cre	0.47 mg/dL	CK	34 U/L
Mono	5.90%	AST	16 U/L	CRP	≤ 0.10 mg/dL
RBC	562 × 10^4^ /µL	ALT	15 U/L	SAA	≤ 2.0 mg/L
Hb	10.4 g/dL	ALP	83 U/L		
Hct	33.2%	γ-GTP	15 U/L		
Plt	20.5 × 10^4^ /µL				

SAA, Serum amyloid A

## Discussion

In the early 21st century, it has become evident that the underlying mechanism of CAPS is the gain-of-function variants in *NLRP3*, which belongs to nucleotide-binding oligomerization domain-like receptor family. These variants lead to excessive release of interleukin-1β (IL-1β), a strikingly proinflammatory cytokine [7]. In the nucleus, the *NLRP3* gene is actively transcribed by NF-κB, which is stimulated by pathogen-associated molecular pattern molecules (PAMPs) derived from bacteria, viruses and lipopolysaccharides (LPS), as well as damage-associated molecular pattern molecules (DAMPs) derived from ATP, uric acid and calcium pyrophosphate. As a consequence, NLRP3 protein forms an inflammasome with apoptosis-associated speck-like protein containing a CARD (ASC) and other components, which activates pro-caspase 1 to caspase 1 and stimulates IL-1β. In patients with CAPS, this pathway is repeatedly activated due to gain-of-function variants of *NLRP3*, resulting in recurrent inflammation and various symptoms, including periodic fever, nonpruritic urticaria-like rash, sensorineural hearing loss, headaches and fatigue.

According to Infevers, a website providing a genetic database for autoinflammatory diseases (https://infevers.umai-montpellier.fr/web/), there are currently over 300 sequence variants identified in *NLRP3* gene. The p.Ile257Met variant identified in our family is a novel one corresponding to “Uncertain Significance” (PM2 and PP4) in ACMG guidelines [8], while we have not confirmed the gain-of-function phenotype in the variant. To confirm a gain-of-function phenotype for the p.Ile257Met variant, further testing is necessary.

GI symptoms are generally not the predominant manifestations of CAPS. However, Kuemmerle-Deschner et al. [5] reported that the GI symptoms such as abdominal pain, diarrhea, constipation, nausea, vomiting occur more frequently in CAPS patients with low penetrance *NLRP3* variants, which result in less inflammasome activation, less caspase activation and less IL-1β production. On the basis of the clinical features of our family pedigree, the p.Ile257Met variant appears to be a low penetrance variant associated with GI symptoms in Japanese patients with CAPS.

There seems to be four possible mechanisms underlying the abdominal symptoms in patients with CAPS. They are (1) serositis, (2) irritable bowel syndrome (IBS), (3) IBD and (4) secondary amyloidosis [9–17]. Among those mechanisms, serositis associated with NLRP3 inflammasome activation through elevated IL-1β production seems to be the most likely, because serositis has been shown to occur in patients with FMF, which is another IL-1β-mediated autoinflammatory disease, even in the absence of abnormalities on CT [18–20]. However, IBS should be considered as a possible contributing factor to her abdominal symptoms.

In our case, the history of sensorineural hearing loss triggered the diagnosis of CAPS. It has been reported that 62% of CAPS patients experience sensorineural hearing loss, and cochlear enhancement on FLAIR-MRI sequences was observed in 87% of CAPS patients with sensorineural hearing loss [21]. This enhancement, indicating the breakdown of the labyrinth-blood barrier of the cochlea, is presumably caused by leakage of inflamed microvessels, leading to cochlear tissue damage [22] and ultimately irreversible sensorineural hearing loss, if left untreated [23, 24]. In this case, the patient’s sensorineural hearing loss persists, presumably due to the prolonged interval of approximately 15 years between its onset and the initiation of treatment.

Wengrower et al. [25] reported that 38% of IBD patients exhibit hearing loss as an extra-intestinal manifestation (EIM). Furthermore, it has been reported that the average hearing thresholds are higher in IBD patients, particularly in those with ulcerative colitis, when compared to healthy people [26, 27]. In monogenic IBD, certain variants are known to cause high-frequency sensorineural hearing loss, such as *STXBP3* variants [28]. Because hearing loss in IBD often progresses slowly and may be asymptomatic in its early stages [27], active audiological assessment should be considered in patients with suspected CAPS or active IBD.

In conclusion, our experience suggests that CAPS should be considered in the differential diagnosis of IBD-like conditions in adolescents. Thus, it seems to be necessary to understand extra-gastrointestinal complications of CAPS. Also, there may be a need to regard the disease as a differential diagnosis of monogenic IBD.
